# Adverse childhood experiences are associated with development of psychological resilience among older adults

**DOI:** 10.1093/geroni/igaf122.4062

**Published:** 2025-12-31

**Authors:** Chenguang Du, Laura Sands, Yan Yuan, Yuewen Zhang, Caiwen Li, Benjamin Katz

**Affiliations:** Northwest Normal University, Lanzhou, Gansu, China (People’s Republic); Virginia Tech, Blacksburg, Virginia, United States; Northwest Normal University, Lanzhou, Gansu, China (People’s Republic); Northwest Normal University, Lanzhou, Gansu, China (People’s Republic); Northwest Normal University, Lanzhou, Gansu, China (People’s Republic); Virginia Tech, Blacksburg, Virginia, United States

## Abstract

**Background:**

A growing body of research suggests that adverse childhood experiences (ACE; e.g. physical abuse) are associated with mental and physical health in young and middle adulthood. However, less is known about how ACEs are linked to psychological resilience in older adults. This study explored the long-term impact of childhood adversity on psychological resilience (PR) trajectories among older adults, drawing on the framework of Cumulative Advantage and Disadvantage (CAD) theory.

**Method:**

Data include 9,069 respondents aged 65 and above from 2006 to 2020 of the Health and Retirement Study (HRS). We first employed the group-based trajectory modeling to classify heterogeneity in PR’s development over time. Next, a multinomial logistic regression was conducted using four different ACE items including: school retention, physical abuse, parental substance abuse, and trouble with police as the main predictors, with covariates (e.g. age, gender, marital status, and education) controlled in the model.

**Result:**

The analyses revealed 3 distinct trajectory groups: one group with stable resilience, and two groups with either “sharp” decline or “gradual” decline. In addition, after adjusting for covariates, we found that of the four ACE items, only experiences of physical abuse and parental substance abuse were significantly associated with the reduced likelihood of maintaining a stable trajectory on PR.

**Conclusion:**

These findings suggest that early adverse experiences may influence trajectories of psychological resilience well into old age. The results underscore the importance of adopting trauma-informed approaches in resilience-based intervention, emphasizing early identification and intervention for individuals with histories of childhood maltreatment.

